# Association of rhinovirus and potentially pathogenic bacterial detections in the first 3 months of life with subsequent wheezing in childhood

**DOI:** 10.1002/ppul.26667

**Published:** 2023-09-06

**Authors:** Mari D. Takashima, Keith Grimwood, Peter D. Sly, Stephen B. Lambert, Robert S. Ware

**Affiliations:** ^1^ School of Medicine and Dentistry, Menzies Health Institute Queensland Griffith University Gold Coast Queensland Australia; ^2^ Departments of Infectious Diseases and Paediatrics Gold Coast Health Gold Coast Queensland Australia; ^3^ Children's Health and Environment Program, Child Health Research Centre The University of Queensland South Brisbane Queensland Australia; ^4^ Australian Infectious Diseases Research Centre The University of Queensland St Lucia Queensland Australia; ^5^ UQ Centre for Clinical Research The University of Queensland Brisbane Queensland Australia; ^6^ National Centre for Immunisation Research and Surveillance of Vaccine Preventable Diseases Sydney New South Wales Australia

**Keywords:** asthma, children, respiratory bacteria, Rhinovirus, wheeze

## Abstract

**Objective:**

Airway interactions between viruses, especially rhinoviruses, and potentially pathogenic bacteria (PPB; *Streptococcus pneumoniae*, *Haemophilus influenzae*, and *Moraxella catarrhalis*) in early infancy may increase the risk of subsequent wheezing and asthma. We evaluated the association between rhinovirus and PPB in the first 3 months of life and wheezing episodes before age 2 years and asthma at age 5–7 years.

**Methods:**

An Australian community‐based birth cohort of healthy children involved parents collecting nasal swabs weekly and completing symptom diaries daily until age 2 years. In a follow‐up subset, asthma diagnosis was assessed annually until age 7 years. Swabs were analyzed by real‐time polymerase chain reaction assays. Children were included if they returned symptom diaries beyond age 3 months (wheeze) or were reviewed at age 5–7 years (asthma).

**Results:**

1440 swabs were returned by 146 children in the first 3 months of life. Wheeze and asthma outcomes were recorded for 146 and 84 children, respectively. Each additional week of rhinovirus detection increased the incidence of wheezing before age 2 years by 1.16 times (95% confidence interval [CI]: 0.99–1.35). There were no significant associations between bacteria and wheeze. Each additional week with *H. influenzae* increased the odds of asthma at age 5–7 years by 135% (odds ratio: 2.35, 95% CI: 0.99–5.58). No significant interaction was observed between rhinovirus and PPB for wheezing or asthma.

**Conclusion:**

Early life rhinovirus infection was associated with wheezing before age 2 years and *H. influenzae* with asthma by age 5–7 years. Microbes may play an etiologic role in wheezing and asthma, warranting further study.

## 1 INTRODUCTION

Acute lower respiratory infections (ALRIs) are the leading cause of acute wheezing episodes in young children. It is possible that ALRIs in early life may injure the developing lungs, adversely affecting airway structure and function, increasing the risk of recurrent wheezing episodes and a later diagnosis of asthma. Cohort studies have reported an association between ALRI during infancy and impaired lung function in later childhood.^1^, ^2^ A population‐based, data linkage study from Western Australia found a positive dose–response association between the number and duration of hospitalizations for ALRIs in the first 12 months of life and subsequent respiratory‐related hospitalizations, including for asthma, after 3 years of age.^3^ However, it is unclear whether this association is causal. In particular, it is unknown whether ALRI is an independent risk factor for later wheeze or a marker of pre‐existing structural and immunological abnormalities leading to later wheeze.

Viral infections, including those caused by rhinoviruses, are implicated in the development of wheezing and asthma in children.^4^, ^5^, ^6^ Rhinoviruses are the most common viral pathogens in young children living in high‐income countries and are detected in up to 67% of acute respiratory infections.^7^, ^8^, ^9^ There are three rhinovirus species: A, B, and C, with type C associated with more severe ALRI and asthma.^10^, ^11^ In a birth cohort study conducted in the United States, rhinovirus was detected in 48% of wheezing illnesses among children up to 3 years of age. Those who had rhinovirus detected when they wheezed had a higher risk of developing asthma at age 6 years compared to children who did not wheeze when either rhinovirus or respiratory syncytial virus were detected (odds ratio [OR]: 9.8, 95% confidence interval [CI]: 4.3–22.0).^6^ An Australian birth cohort study reported that children with rhinovirus‐related wheezing ALRI episodes in the first year of life had increased odds of persistent wheezing (OR: 2.9; 95% CI: 1.2–7.0) and asthma (OR: 2.9; 95% CI: 1.2–7.1) at age 5 years.^12^

Colonizing upper airway bacteria may also result in aberrant programming of the host immune system and a predisposition to recurrent wheezing and asthma.^13^ Previous studies have reported colonization with potentially pathogenic bacteria (PPB), including bacteria most commonly detected in the upper respiratory tract of Australian infants (*Streptococcus pneumoniae*, *Haemophilus influenzae*, and *Moraxella catarrhalis*),^14^ is associated with an increased risk of ALRI^15^, ^16^ and wheeze in young children,^17^, ^18^ and asthma in later childhood.^19^ The first 1–3 months of life is a critical time period for airway microbiota development, and birth cohort studies have reported colonization by PPB during this period was associated with increased risk of recurrent wheezing and asthma.^16^, ^19^, ^20^, ^21^ A Finnish birth cohort study observed that early maturation of the nasopharyngeal microbiota with *Moraxella* genera and accompanying instability following incursions by *H. influenzae* and *S. pneumoniae*, increased the risk of developing asthma when compared with infants possessing a persistent *Moraxella* dominant nasopharyngeal profile.^21^

The present study aimed to use data from an Australian community‐based, healthy birth cohort to evaluate the association between rhinoviruses and PPB (*S. pneumoniae*, *M. catarrhalis*, and *H. influenzae*) in the upper airway during the first 3 months of life and (i) wheeze in the first 2 years of life; and (ii) asthma at age 5–7 years. We also evaluated whether there was evidence of an interaction between rhinoviruses and PPB.

## 2 METHODS

### 2.1 Study subjects and setting

The Observational Research in Childhood Infectious Diseases (ORChID) study (clinicaltrials.gov: NCT01304914) was a community‐based, birth cohort of acute respiratory infections in healthy children during the first 2 years of life.^22^ ORChID study participants lived in the subtropical city of Brisbane, Australia. Mothers were recruited from antenatal clinics at one of two metropolitan hospitals (one private and one government‐funded) between September 2010 and October 2012. Healthy children born at 36–42 weeks' gestation without congenital abnormalities or underlying chronic disorders were enrolled in the study. Parents provided informed consent for their child's participation shortly after birth. During the ORChID study, parents kept a daily symptom diary for their child, and collected weekly nasal swab specimens. Children exited the study when their parents stopped returning study material, or at their second birthday, whichever occurred earlier. At the end of their involvement with the ORChID project, children and their parents/caregivers were invited to participate in an extension study, the Early Life Lung Function (ELLF) study.^23^ This required an annual review by research staff between ages 3–7 years and included completion of a standardized respiratory health questionnaire. The Royal Brisbane and Women's Hospital (HREC/10/QRBW125) Human Research Ethics Committee (HREC) approved the ORChID study. The Children's Health Queensland (HREC/10/QRCH/16 and HREC/13/QRCH/156) and The University of Queensland (2010/HE00820 and 2013/HE001291) HRECs approved the ORChID and ELLF studies.

### 2.2 Recording of sociodemographic characteristics and illness episodes

At enrollment, parents provided sociodemographic and health characteristics, including pregnancy and birth details.^24^ They received a digital thermometer and diary cards to complete daily, listing pre‐defined respiratory symptoms and diagnoses in tick‐box format. Parents were taught to recognize respiratory symptoms, including wheezing and shortness of breath. When symptoms occurred, parents recorded healthcare visits in a separate illness‐burden diary. Both diaries were returned to the research team monthly by mail.

During the ORChID study, parents were interviewed by telephone every 3 months to update information on feeding practices and childcare arrangements. Exclusive breastfeeding occurs when the child was breastfed without taking milk formula or solids.^22^ Childcare was categorized as formal (regulated care outside the child's home) and informal (nonregulated care by family or friends). Vaccination data were captured from the Australian Immunization Register.

### 2.3 Respiratory specimen collection and testing

In the ORChID study, bilateral anterior nasal swabs were collected at birth and thereafter weekly by parents using a single swab regardless of symptoms. All swabs were collected using a plastic‐shaft, rayon‐budded swab and inserted into a transport tube with a foam pad reservoir soaked with viral transport medium (Virocult MW950, Medical Wire & Equipment). The median interval between swabs was 7 days (interquartile range [IQR]: 7–12). Collected swabs were surface‐mailed to the study laboratory (received median [IQR] 3 days [2–3] after collection), where they were stored at −80°C. Swabs were batch‐tested for 17 respiratory viruses, as well as *S. pneumoniae*, *M. catarrhalis*, and *H. influenzae* by previously validated real‐time polymerase chain reaction (PCR) assays.^9^, ^14^ All virus and bacterial detections with PCR cycle threshold (Ct) values < 40 were considered positive. Specimen quality was assessed by testing for a marker of human genomic DNA, endogenous retrovirus‐3 (ERV‐3). As rhinovirus was by far the predominant virus detected in the first 3 months of life,^9^ analyses of viruses in this study were restricted to rhinovirus detections. Rhinovirus genotyping was achieved by amplifying variable region VP4/VP2 genes. The PCR products were purified using the QIAquick PCR purification kit (Qiagen) and were then submitted for DNA sequencing to the Australian Genome Research Facility (The University of Queensland). Phylogenetic analysis was performed on a 230‐bp section of the sequenced VP4/VP2 region (see Supporting Information: Methods).

### 2.4 Wheeze and asthma

The primary outcomes were wheezing episodes reported between 3 and 24 months of life, and asthma at age 5–7 years. The presence of wheeze was extracted from the parent‐completed daily symptom and illness‐burden diaries. Children who supplied diary data beyond the age of 3 months were included in this analysis. At ages 5–7 years, parents were asked whether their child had ever received a diagnosis of asthma from a doctor, or if they had used an inhaled beta‐2 agonist or inhaled corticosteroid asthma medication in the previous 12 months.^25^

### 2.5 Analysis

The association between rhinovirus and PPB detections in the first 3 months of life and wheeze in the first 2 years of life was investigated using Poisson regression models offset by the natural logarithm of the number of diary days returned. The association between rhinovirus and PPB detections and asthma at age 5–7 years was analyzed using logistic regression models. First, we constructed a single regression analysis with four main effects (rhinovirus, *S. pneumoniae*, *M. catarrhalis*, and *H. influenzae*). This model, called the “Rhinovirus and Bacteria Model,” describes the contribution of each variable to the outcome after adjusting for the other three covariables. Second, the “Full Adjusted Model” included rhinovirus and the three PPBs as main effects, and potentially confounding variables as covariables. The covariables selected were based upon the results of a recent systemic review,^26^ expert clinician opinion, and directed acyclic graphs (Supporting Information: E‐images 1 and 2). These included: season of birth, childcare attendance, tobacco smoke exposure at birth, maternal (for wheeze outcome only) and parental (for asthma outcome only) history of asthma, gestational age at birth, exclusive breastfeeding during the first 3 months of life (for wheeze outcome only), cesarean birth and an older child in the household at birth. To examine the sensitivity of models, we constructed “Single Pathogen Models,” which are individual models each with a single main effect, and “Single Pathogen Adjusted Models,” which are the individual models adjusted for the covariables listed above. Possible interaction effects were examined by adding the interaction term, assessing the statistical significance (alpha set at 0.05) and comparing the magnitude of the main exposure effect sizes with and without the interaction term. We examined the sensitivity of the timing of exposure by re‐running models examining rhinovirus and PPB detections in the first 4 weeks and first 6 months of life.^19^ Additional analyses considering each of the three rhinovirus species in the first 3 months were also performed.^4^ Missing values were not imputed. All analyses were conducted using Stata statistical software v13 (StataCorp).

## 3 RESULTS

### 3.1 Study population

Overall, 158 children were included in the full ORChID study, with 146 supplying diary data for the wheeze outcome and 84 providing asthma data at age 5–7 years. Characteristics of children in the full ORChID cohort were generally similar to those included in these analyses, except for a higher prevalence of tobacco smoke exposure at birth in excluded children (5/12 [41.7%]) compared to children included in the analysis (14/144 [9.7%]) for wheeze outcome (Supporting Information: E‐Table 1).

Sociodemographic and clinical characteristics are described in Table 1. Of the 146 children who provided wheezing outcomes, most were born between 39 and 41 weeks' gestation (*n* = 114 [78.1%]) into a single‐child household (*n* = 95 [65.1%]) where at least one parent had asthma (*n* = 66 [45.2%]). Four children were attending childcare at the age of 3 months. Children providing an asthma outcome (*n* = 84) had similar characteristics to those providing wheeze outcomes (Table 1).

**Table 1:** Sociodemographic characteristics of ORChID (*N* = 146) and ELLF (*N* = 84) children providing wheeze or asthma outcome data.

	ORChID (*N* = 146), *N* (%)	ELLF (*N* = 84), *N* (%)
Sex (male)	70 (47.9)	40 (47.6)
Season of birth		
Summer (December–February)	40 (27.4)	24 (28.5)
Fall (March–May)	25 (17.1)	15 (17.9)
Winter (June–August)	40 (27.4)	22 (26.2)
Spring (September–November)	41 (28.1)	23 (27.3)
Vaginal delivery	98 (67.1)	55 (65.5)
Gestational age at birth		
36–38 weeks	32 (21.9)	15 (17.8)
39–41 weeks	114 (78.1)	69 (82.1)
Family history		
Either parent has asthma	66 (45.2)	40 (47.6)
Mother has asthma	40 (27.6)	22 (26.2)
Household smoke exposure at birth	(n = 144)	(n = 84)
Yes	14 (9.7)	10 (11.9)
Older child(ren) in house at birth	51 (34.9)	29 (34.5)
Maternal education status		
University degree	94 (64.8)	55 (65.5)
Diploma/certificate	35 (24.1)	21 (25.0)
Secondary school	16 (11.0)	8 (9.5)
Mode of feeding		
Exclusive breastfeeding until at least 3 months of age	99 (67.8)	58 (69.1)
Childcare attendance at 3 months^a^		
No childcare	138 (97.2)	80 (95.2)
Formal and informal childcare	4 (2.8)	4 (4.8)
Childcare attendance at 6 months^a^		
No childcare	102 (76.7)	60 (72.3)
Formal and informal childcare	31 (23.3)	23 (27.7)
Pneumococcal conjugate vaccine doses^b^		
6 weeks	142 (97.2)	81 (96.4)
4 months	141 (96.5)	83 (98.8)
6 months	137 (93.8)	83 (98.8)

Abbreviations: ELLF, Early Life Lung Function; ORChID, Observational Research in Childhood Infectious Disease.

^a^ Formal childcare was defined as outside homecare from a regulated childcare service, while informal care comprised nonregulated care by family or friends.

^b^ Pneumococcal conjugate vaccine was administered as a three‐dose primary course without a booster in the second year of life.

**Figure 1:**
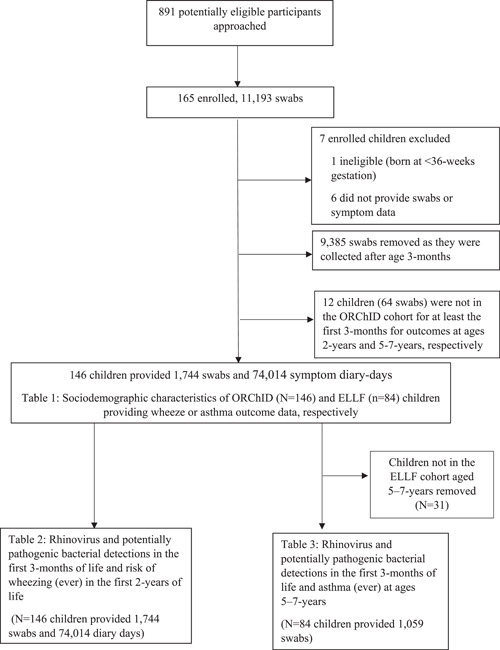
Flow chart of children and nasal swabs in the Observational Research in Childhood Infectious Diseases study.

The 146 children who provided data for either outcome returned 1774 swabs during their first 3 months of life (Figure 1). Of 1400 swabs with an ERV3 value, 463 (33.1%) recorded a virus and/or PPB detections, compared with 54/344 (15.7%) of swabs with no ERV3 value recorded. The number of children returning swabs each week for their first 6 months of life (6 months chosen because of the sensitivity analysis) is displayed in Supporting Information: E‐Image 3. There were 74,014 diary‐days returned (79% of maximum possible).

Rhinovirus and PPB were identified progressively from the first week of life (Supporting Information: E‐Image 4). Rhinovirus was detected at least once in 71/146 (48.6%) children who provided diary data in the first 3 months of life (Supporting Information: E‐Table 2). In addition, *S. pneumoniae*, *M. catarrhalis*, and *H. influenzae* were detected in 72 (49.3%), 41 (28.1%), and 16 (10.9%) children, respectively, during the same period. Rhinovirus was by far the most frequently detected virus in the first 3 months of life (151 detections; 8.1% of 1774 swabs returned; Supporting Information: E‐Table 2). Of the 151 rhinovirus detections, 40 (26.5%) were rhinovirus‐A, 24 (15.9%) were rhinovirus‐B, 37 (24.5%) were rhinovirus‐C, and 50 (33.1%) were unable to be typed (Supporting Information: E‐Table 3). There were 91 discrete rhinovirus episodes, of which most were asymptomatic (*n* = 70 [76.9%]), including all involving rhinovirus‐B (Supporting Information: E‐Table 3). When rhinovirus was detected, compared to when it was not detected, PPBs were more likely to be detected from the same swab (*S. pneumoniae*: OR: 2.30 [95% CI: 1.56–3.40]; *M. catarrhalis*: OR: 3.88 [95% CI: 1.93–4.30]; and *H. influenzae* [OR: 4.86; 95% CI: 2.48–9.51], Supporting Information: E‐Table 4).

### 3.2 Rhinovirus and potentially pathogenic bacterial detections in the first 3 months and wheeze in the first 2 years of life

There were 48/146 (32.9%) children whose parents reported they wheezed between ages 3 and 24 months. Each extra week of rhinovirus infection in the first 3 months of life led to a 1.16 times increase in wheezing within the first 2 years of life, after adjusting for the presence of PPB (incident rate ratio [IRR]: 1.16; 95% CI: 0.99–1.35; Table 2, Supporting Information: E‐Table 5). Point estimates were similar after adjusting for potentially confounding variables (IRR: 1.14; 95% CI: 0.95–1.37; Supporting Information: E‐Image 5, Table 2). The associations between *S. pneumoniae*, *M. catarrhalis*, and *H. influenzae* and wheezing were IRR (95% CI): 1.02 (0.89–1.15), 1.01 (0.90–1.13), and 1.04 (0.77–1.40), respectively. No significant interaction effect was observed between rhinovirus and PPB.

**Table 2:** Rhinovirus and potentially pathogenic bacterial detections in the first 3 months of life and risk of wheezing (ever) in the first 2 years of life (*N* = 146).

Pathogens	No wheeze (ever)	Wheeze (ever)	Rhinovirus and bacteria model^b^	Full adjusted model^c^
	*N* = 98	*N* = 48	*N* = 146	*N* = 144
	Mean (SD)^a^	Mean (SD)^a^	IRR (95% CI)	IRR (95% CI)
Rhinovirus	0.82 (1.45)	1.48 (1.94)	1.16 (0.99–1.35)	1.14 (0.95–1.37)
*Streptococcus pneumoniae*	1.64 (2.64)	2.02 (2.99)	1.01 (0.91–1.14)	1.02 (0.89–1.15)
*Moraxella catarrhalis*	1.35 (3.04)	1.56 (3.13)	0.99 (0.90–1.10)	1.01 (0.90–1.13)
*Haemophilus influenzae*	0.27 (0.99)	0.35 (1.23)	1.04 (0.78–1.37)	1.04 (0.77–1.40)

Abbreviations: CI, confidence interval; IRR, incident rate ratio; SD, standard deviation.

^a^ Mean number of weeks a pathogen was detected in the first 3 months of life.

^b^ A Poisson regression analysis with four main effects (rhinovirus, *S. pneumoniae, M. catarrhalis*, and *H. influenzae*).

^c^ Adjusted for season of birth, maternal asthma history, gestational age, delivery method, tobacco smoke exposure at birth, exclusive breastfeeding during the first 3 months of life, and older child in the household at birth. Childcare attendance at 3 months was omitted as only four children were attending any form of childcare.

When examining the sensitivity of results to the exposure period, the low frequency of pathogen detection in the first 4 weeks of life led to imprecise associations with wide CIs (Supporting Information: E‐Table 6). When detections in the first 6 months of life were considered, findings were similar to the main analysis but with more precision (IRR: 1.13; 95% CI: 1.04–1.24). When rhinovirus species were analyzed separately at age 3 months, rhinovirus‐B and rhinovirus‐C had stronger associations on wheeze (IRR: 1.10; 95% CI: 0.86–1.41 and IRR: 1.19; 95% CI: 0.96–1.48, respectively) than rhinovirus‐A (IRR: 0.88; 95% CI: 0.59–1.31; Supporting Information: E‐Table 7).

### 3.3 Rhinovirus and potentially pathogenic bacterial detections in the first 3 months of life and asthma at 5–7 years

There were 29/84 (34.5%) children identified as having asthma at age 5–7 years. *H. influenzae* detections were positively associated with asthma in the rhinovirus and bacteria model (OR: 2.35; 95% CI: 0.99–5.58; Table 3, Supporting Information: E‐Image 6, Supporting Information: E‐Table 8). The association was similar after adjusting for potentially confounding variables (OR: 2.25; 95% CI: 0.88–5.73). The associations between rhinovirus, *S. pneumoniae*, and *M. catarrhalis* with asthma at ages 5–7 years were OR: 1.05 (95% CI: 0.71–1.57), OR: 0.73 (95% CI: 0.51–1.06), and OR: 1.09 (95% CI: 0.86–1.39), respectively. No interaction effects between rhinovirus and PPB were observed.

**Table 3:** Rhinovirus and potentially pathogenic bacterial detections in the first 3 months of life and asthma (ever) at ages 5–7 years (*N* = 84).

Pathogen	No asthma (ever)	Asthma (ever)	Rhinovirus and bacteria model^b^	Full adjusted model^c^
	*N* = 55	*N* = 29	*N* = 84	*N* = 84
	Mean (SD)^a^	Mean (SD)^a^	OR (95% CI)	OR (95% CI)
Rhinovirus	0.98 (1.58)	1.07 (1.46)	1.00 (0.74–1.36)	1.05 (0.71–1.57)
*Streptococcus pneumoniae*	1.65 (2.57)	1.21 (2.13)	0.79 (0.59–1.05)	0.73 (0.51–1.06)
*Moraxella catarrhalis*	1.44 (2.98)	1.66 (3.65)	0.99 (0.84–1.15)	1.09 (0.86–1.39)
*Haemophilus influenzae*	0.07 (0.33)	0.45 (1.43)	2.35 (0.99–5.58)	2.25 (0.88–5.73)

Abbreviations: CI, confidence interval; OR, odds ratio; SD, standard deviation.

^a^ Mean number of weeks a pathogen was detected in the first 3 months of life.

^b^ A single logistic regression analysis with four main effects (rhinovirus, *S. pneumoniae, M. catarrhalis*, and *H. influenzae*).

^c^ Adjusted for season of birth, parental asthma history, gestational age, delivery method, tobacco smoke exposure at birth, exclusive breastfeeding during the first 3 months of life, and older child in the household at birth. Childcare attendance at 3 months was omitted as only four children were attending any form of childcare.

Sensitivity analyses considering detections during the first 6‐months showed similar effect sizes to the main results for rhinovirus, *S. pneumoniae* and *M. catarrhalis* (Supporting Information: E‐Table 9). However, the association with *H. influenzae* was attenuated (OR: 1.18, 95% CI: 0.96–1.45). When each rhinovirus species at age 3 months was included in the “rhinovirus and bacteria model,” *H. influenzae* had consistently strong associations with asthma (Supporting Information: E‐Table 10).

## 4 DISCUSSION

In this community‐based birth cohort of healthy Australian children, each additional week of rhinovirus detections in the first 3 months of life was associated with an increased risk of reporting wheezing episodes in the first 2 years of life. However, rhinovirus infections in the first 3 months of life were not associated with asthma at ages 5–7‐years. Although PPB did not have strong associations with wheezing, *H. influenzae* detections in early life increased asthma OR point estimate at age 5–7 years by 135%. No interaction effects between rhinovirus and PPB were observed for either wheezing or asthma.

While many studies have reported the associations between rhinovirus‐induced wheezing illness and recurrent wheezing^27^, ^28^, ^29^ and asthma,^6^, ^12^, ^29^ it is not clear whether any rhinovirus infections (symptomatic or asymptomatic) in early life are associated with wheezing in the first 2 years of life.^30^ A potential rationale for the positive dose–response relationship with wheezing in the first 2 years of life in this study is that children who encounter a higher number of rhinovirus infections during the period from birth to the first 3 months of life may have an increased likelihood of subsequent rhinovirus infections until 2 years of age.^31^ Consequently, repeated rhinovirus infections may result in a higher incidence of wheezing episodes induced by rhinovirus, which also results in a higher incidence of childhood asthma.

*H. influenzae* was associated with an increased risk of asthma, however, due to the small size of our sample, our findings should be interpreted with caution. Nevertheless, recent research using the clustering approach in a 17‐center United States cohort study of 921 infants hospitalized with bronchiolitis identified three immunophenotypes, suggesting a heterogeneous immune response.^32^ Those at the greatest risk of asthma had a history of eczema, bronchiolitis associated with rhinovirus, higher peripheral blood eosinophil counts, and an upper airway microbiome dominated by *H. influenzae* and *M. catarrhalis*, suggesting an altered mucosal immune response to these PPB. The presence of these PPBs could be part of a more complex host–pathogen relationship and immunophenotype associated with asthma.

Birth cohort studies have reported mixed results on the early detection of PPB and its relationship to subsequent asthma. In the Copenhagen Prospective Study on Asthma in Childhood (COPSAC) birth cohort of 321 neonates from mothers with asthma, colonization of the airways with *S. pneumoniae*, *H. influenzae*, *M. catarrhalis*, either alone or in combination in asymptomatic neonates at 1 month of age was associated with the development of asthma by age 5 years (OR: 4.57, 95% CI: 2.18–9.57).^19^ However, in the Childhood Origins of Asthma birth cohort study of 289 children, a *Staphylococcus*‐dominant microbiome in the first 6 months of life was associated with an increased risk of recurrent wheezing by age 3 years and asthma that persisted throughout childhood.^33^ In the Childhood Asthma Study (of 244 children with high risk of asthma sensitization, early asymptomatic *Streptococcus* colonization in the first 2 months of life was significantly associated with chronic wheeze at age 5 years (OR: 3.8, 95% CI: 1.3–12.0).^16^ The authors reported that *Haemophilus* was very rare in nasopharyngeal samples from healthy infants, and *Moraxella* colonization was established later during infancy, which the authors suggested may be due to Perth's warm Mediterranean‐like climate, and contrasts with the results in healthy infants from the warm humid subtropical climate of South‐East Queensland.

The viral and bacterial interaction on recurrent wheezing was not detected in two other cohort studies. In the COPSAC birth cohort, upper airway viruses (OR: 2.8, 95% CI: 1.7–4.4) and bacteria (OR: 2.9, 95% CI: 1.9–4.3) detected by PCR assays and conventional culture methods, respectively, were associated with wheezing episodes, but the associations of viruses and bacteria were independent of one another.^17^ Another prospective cohort study compared oropharyngeal swabs from 109 children with an acute wheezing illness and 75 non‐wheezing children attending a tertiary pediatric hospital in Perth, Australia.^34^ In this study, rhinoviruses did not have a significant impact upon bacterial community composition determined by 16S rRNA gene sequencing, and wheezing and viruses were not related to the bacterial community. These results implied there was no interaction between viruses and bacteria.

There are two possible, nonexclusive theories describing susceptibility to wheezing and asthma after early virus and PPB detections in young children. Firstly, host immune programming at birth is skewed toward Th2 responses and developing asthma. Infants with poor innate antiviral defenses develop more severe illnesses during infections with respiratory viruses.^35^ Early and more severe infections could lead to increased damage to the lower airways during a critical growth phase of the lungs, leading to changes in airway structure and function that promote asthma. In the COPSAC birth cohort, children who eventually developed asthma had an aberrant early life immune response evident by increased IL‐5, IL‐13, IL‐17, and IL‐10 production, which might predispose to persistent lower airway resident PPB colonization and result in chronic airway inflammation progressing to asthma.^13^ In addition, rhinovirus‐related hospitalization rates are especially high for infants and children with asthma, which suggests viral infections and their associated induced host inflammatory response might directly injure the lower airways during acute infections.^36^ Alternatively, susceptible children may constitutionally have smaller airways and thus are predisposed to increased airway obstruction leading to recurrent wheezing during ALRIs and subsequently to asthma. This latter theory suggests that children who are born with small airways are also vulnerable to respiratory viral infections like rhinovirus and respiratory syncytial virus, which heighten the risk of ALRI and wheezing.

Strengths of the ORChID study include its longitudinal design and weekly sampling of nasal swabs in a healthy, community‐based birth cohort allowing the analysis of cumulative temporal rhinovirus and PPB detections in the first 3 and 6 months of life. There are, however, several limitations. First, despite sensitive PCR assays, suboptimal swabbing techniques may have missed virus detections. Nevertheless, we have shown that parent‐collected nasal swabs have similar virus detection rates to those obtained by health personnel when employing PCR assays.^37^ Second, the number of ALRI episodes in the first 3 months of life was low in the ORChID cohort, and thus their impact on subsequent wheezing episodes and asthma may have been underestimated. Third, our statistical analysis had suboptimal power for some comparisons due to the low incidence of *H. influenzae* detections. Fourth, we did not serotype the *S. pneumoniae* isolates. Fifth, unbiased next‐generation sequencing to examine alterations in microbial community profiles during the first 3 months of life was not performed. However, low bacterial DNA loads in nasal swabs limit these studies to 16S rRNA gene sequencing and often organization taxonomic unit discrimination does not go beyond the genus level. Instead, we included all three PPBs in the “rhinovirus and bacteria model” to mimic the nature of co‐existing bacteria found consistently in the upper airways by conventional culture‐based methods. Sixth, we used an epidemiologic definition for the diagnosis of asthma, and it relied upon assessment by a physician and was not supplemented by at least two objective measures, such as fractional exhaled nitric oxide values, demonstration of airway hyperresponsiveness or a positive bronchodilator response as recommended by the European Respiratory Society in children aged 5–16 years.^38^ This study was conducted before the European guidelines were published, and we followed the Australian guidelines that recommended clinical assessment as the most reliable means of diagnosing asthma in this age group.^39^ Seventh, mainly because of low viral loads, one third of rhinoviruses were unable to be sequenced. However, this is common in community‐based studies where mild and asymptomatic infections are encountered.^40^, ^41^, ^42^ Eighth, we did not test the atopic status of children. Future studies should consider the relationship between atopy and early sensitization and pathogen detections. Finally, as often occurs with intense studies of this nature, the results may not generalize to children from other backgrounds and environmental settings. Our cohort had a high rate of parental asthma which may reduce the generalizability of our findings.

In conclusion, early rhinovirus infections were associated with increased odds of wheezing in the first 2 years of life, and early *H. influenzae* colonization was associated with increased odds of asthma between 5 and 7 years of age. The underlying mechanisms for developing recurrent wheezing and asthma after rhinovirus or PPB detections in young children remain unclear. There may be different immune responses to these microbes between atopic children, those with constitutional small airways, and otherwise healthy children. Future research should explore whether recurrent wheezing and asthma are from inherited structural airway abnormalities, a complex interplay between rhinoviruses and abnormal host immune responses to airway colonization by PPB or viral infections, or all these factors.^43^

## AUTHOR CONTRIBUTIONS

**Mari D. Takashima**: Conceptualization; data curation; formal analysis; methodology; project administration; software; validation; visualization; resources; writing—original draft; writing—review and editing. **Keith Grimwood**: Conceptualization; methodology; investigation; supervision; writing—review and editing; writing—original draft; funding acquisition; resources; project administration. **Peter D. Sly**: Conceptualization; methodology; investigation; funding acquisition; supervision; writing—review and editing; writing—original draft; resources; project administration. **Stephen B. Lambert**: Conceptualization; methodology; investigation; funding acquisition; supervision; writing—review and editing; writing—original draft; resources; project administration. **Robert S. Ware**: Conceptualization; methodology; investigation; funding acquisition; supervision; writing—review and editing; validation; visualization; software; formal analysis; data curation; resources; project administration.

## CONFLICT OF INTEREST STATEMENT

The authors declare no conflict of interest.

## ETHICS STATEMENT

The Royal Brisbane and Women's Hospital (HREC/10/QRBW125) Human Research Ethics Committee (HREC) approved the ORChID study. The Children's Health Queensland (HREC/10/QRCH/16 and HREC/13/QRCH/156) and The University of Queensland (2010/HE00820 and 2013/HE001291) HRECs approved the ORChID and ELLF studies.

## Supporting information

Supporting information.

## Data Availability

The data that support the findings of this study are available on request from the corresponding author.
