# Supplementary material for: Association of rhinovirus and potentially pathogenic bacterial detections in the first 3 months of life with subsequent wheezing in childhood
Source: Pediatr Pulmonol. 2023 Sep 6;58(12):3428–36. doi: 10.1002/ppul.26667 (PMC10947429; doi:10.1002/ppul.26667)
Supplement: Supplementary file 1 — Supporting information. [file PPUL-58-3428-s001.docx]

**SUPPLEMENTARY TABLES AND FIGURES**

**Association of rhinovirus and potentially pathogenic bacterial detections in the first
3-months of life with subsequent wheezing in childhood**

Mari D. Takashima MEpi,^1^ Keith Grimwood FRACP MD,^1,2^ Peter D. Sly FRACP DSc,^3,4^ Stephen B. Lambert MBBS PhD,^5,6^ Robert S. Ware PhD.^1^

^1^School of Medicine and Dentistry, and the Menzies Health Institute Queensland, Griffith University, Gold Coast, Queensland, Australia; ^2^Departments of Infectious Diseases and Paediatrics, Gold Coast Health, Gold Coast, Queensland, Australia; ^3^Children’s Health and Environment Program, Child Health Research Centre, The University of Queensland, South Brisbane 4101, Queensland, Australia; ^4^Australian Infectious Diseases Research Centre, The University of Queensland, St Lucia, Queensland, Australia. ^5^UQ Centre for Clinical Research, The University of Queensland, Herston, Queensland, Australia; ^6^National Centre for Immunisation Research and Surveillance of Vaccine Preventable Diseases, Westmead, New South Wales, Australia.

**Corresponding author:**

Mari Takashima

School of Medicine and Dentistry, Griffith University Nathan campus

Nathan, Queensland 4111, AUSTRALIA

Email: [m.takashima@griffith.edu.au](mailto:m.takashima@griffith.edu.au)

Telephone: +61 (07) 3735 5117

**Supplementary Methods**

*Rhinovirus genotyping and phylogenetic analysis*

The rhinovirus variable region VP4/VP2 genes were amplified using a nested PCR assay that comprised two sets of primers^1^. PCR products were purified using the QIAquick PCR purification kit (Qiagen, Australia) and were then submitted for DNA sequencing to the Australian Genome Research Facility (The University of Queensland, Brisbane, Australia).

Phylogenetic analysis was performed on a 230bp section of the sequenced VP4/VP2 region in Geneious 10.2.6 (Biomatters Ltd., Auckland, New Zealand) using the Neighbour-Joining method, Tamura-Nei distance model^2^ and 1000 bootstraps. Sequences shorter than 230bp were excluded from the phylogenetic analysis. Coxsackievirus A11 (accession# JF260917) was used as the outgroup along with reference sequences for HRV-A, HRV-B, and HRV-C (KM362429, FJ445169, and EF077279, respectively). Sequences were dereplicated to represent one genotype per episode in each child, with a replicate sequence being defined as the same genotype occurring within a 7-day period. Evolutionary analyses were conducted in
MEGA X^3^.

**References**

1. Wisdom A, Leitch ECM, Gaunt E, Harvala H, Simmonds P. Screening respiratory samples for detection of human rhinoviruses (HRVs) and enteroviruses: comprehensive VP4-VP2 typing reveals high incidence and genetic diversity of HRV species C. *J Clin. Microbiol*. 2009;47(12):3958-67.
2. Tamura K, Nei M. Estimation of the number of nucleotide substitutions in the control region of mitochondrial DNA in humans and chimpanzees. *Mol Biol Evol*. 1993;10(3):512-26.
3. Kumar S, Stecher G, Li M, Knyaz C, Tamura K. MEGA X: Molecular Evolutionary Genetics Analysis across Computing Platforms. *Mol Biol Evol*. 2018;35(6):1547-9.

## **E-Table 1: Comparison of sociodemographic characteristics of children included and not included in the analyses from the whole ORChID cohort (N=158).**

|  | ORChID  cohort | Wheeze outcomes   (ORChID) | | | Asthma outcomes   (ELLF) | | |
| --- | --- | --- | --- | --- | --- | --- | --- |
|  | **(N=158)**  **N (%)** | **Included**  **(N=146)**  **N (%)** | **Not included**  **(N=12)**  **N (%)** | **p-value**^a^ | **Included**  **(N=84)**  **N (%)** | **Not included**  **(N=74) N (%)** | **p-value**^a^ |
| Sex (Male) | 75 (47.5) | 70 (47.9) | 5 (41.7) | 0.68 | 40 (47.6) | 35 (47.3) | 0.97 |
| Season of birth |  |  |  |  |  |  |  |
| Summer (December-February) | 42 (26.7) | 40 (27.4) | 2 (16.7) | 0.21 | 24 (28.5) | 18 (24.3) | 0.93 |
| Fall (March-May) | 30 (19.0) | 25 (17.1) | 5 (51.7) |  | 15 (17.9) | 15 (20.3) |  |
| Winter (June-August) | 43 (27.2) | 40 (27.4) | 3 (25.0) |  | 22 (26.2) | 21 (28.4) |  |
| Spring (September-November) | 43 (27.2) | 41 (28.1) | 2 (16.7) |  | 23 (27.3) | 20 (27.0) |  |
| Vaginal delivery | 107 (67.7) | 98 (67.1) | 9 (75.0) | 0.58 | 55 (65.5) | 52 (70.3) | 0.52 |
| Gestational age at birth |  |  |  |  |  |  |  |
| 36–38 weeks | 36 (22.8) | 32 (21.9) | 4 (33.3) | 0.37 | 15 (17.8) | 21 (28.4) | 0.12 |
| 39–41 weeks | 122 (77.2) | 114 (78.1) | 8 (66.7) |  | 69 (82.1) | 53 (71.6) |  |
| Family history |  |  |  |  |  |  |  |
| Either parent has asthma | 69 (43.7) | 66 (45.2) | 3 (25.0) | 0.18 | 40 (47.6) | 29 (39.2) | 0.29 |
| Mother has asthma status | 42 (27.8) | 40 (27.6) | 2 (33.3) | 0.09 | 22 (26.2) | 20 (29.9) | 0.62 |
| Household tobacco smoke exposure at birth | (n=156)  19 (12.2) | (n=144)  14 (9.7) | (n=12)  5 (41.7) | <0.01 | (n=84) 10 (11.9) | (N=72)  9 (12.5) | 0.91 |
| Older child(ren) in house at birth | 55 (34.8) | 51 (34.9) | 4 (33.3) | 0.91 | 29 (34.5) | 26 (35.1) | 0.94 |
| Maternal education status |  |  | *1 missing* |  |  | *1 missing* |  |
| University degree | 99 (63.1) | 94 (64.8) | 5 (41.7) | 0.07 | 55 (65.5) | 44 (60.3) | 0.43 |
| Diploma/certificate | 38 (24.2) | 35 (24.1) | 3 (25.0) |  | 21 (25.0) | 17 (23.3) |  |
| Secondary school | 20 (12.7) | 16 (11.0) | 4 (33.3) |  | 8 (9.5) | 12 (16.4) |  |
| Mode of feeding |  |  |  |  |  |  |  |
| Exclusive breastfeeding until at   least age 3-months | 101 (67.3) | 99 (67.8) | 2 (50.0)  *8 missing* | 0.45 | 58 (69.1) | 43 (65.2)  *8 missing* | 0.61 |
| Childcare attendance at age  3-months^b^ (N=142) |  |  |  |  |  | *16 missing* |  |
| No childcare | 138 (97.2) | 138 (97.2) | NA |  | 80 (95.2) | 58 (100.0) | 0.09 |
| Formal and informal childcare | 4 (2.8) | 4 (2.8) | NA |  | 4 (4.8) | 0 (0.0) |  |
| Childcare attendance at age  6-months^b^ (N=133) |  |  |  |  |  | *24 missing* |  |
| No childcare | 102 (76.7) | 102 (76.7) | NA |  | 60 (72.3) | 42 (84.0) | 0.12 |
| Formal and informal childcare | 31 (23.3) | 31 (23.3) | NA |  | 23 (27.7) | 8 (16.0) |  |

Abbreviations: ELLF: Early Life Lung Function; NA: Not applicable; ORChID: Observational Research in Childhood Infectious Disease

^a^Chi-square tests. Significance set at <0.05.

^b^Formal childcare was defined as outside homecare from a regulated childcare service, while informal care comprised non-regulated care by family or friends.

**E-Table 2: Cumulative number and percentages of respiratory virus and potentially pathogenic bacterial detections in first 3- and
6-months life for ORChID study participants included in this study (N=146)**

|  | **3-months of life** | | **6-months of life** | |
| --- | --- | --- | --- | --- |
|  | **N (%)** | | **N (%)** | |
| **Number of swabs returned** | **1774** | | **3185** | |
| **Organisms** | **Children** | **Positive swabs** | **Children** | **Positive swabs** |
| Rhinovirus | 71 (48.6) | 151 (8.1) | 115 (78.8) | 381 (12.0) |
| Human coronavirus | 9 (6.2) | 13 (0.7) | 16 (10.9) | 19 (0.6) |
| Respiratory syncytial virus | 3 (2.1) | 5 (0.3) | 12 (8.2) | 12 (0.4) |
| Human metapneumovirus | 1 (0.7) | 1 (0.1) | 1 (0.7) | 1 (<0.1) |
| Parainfluenza viruses 1–3 | 7 (4.8) | 7 (0.4) | 13 (8.9) | 12 (0.4) |
| Influenza virus | 2 (1.4) | 2 (0.1) | 3 (2.1) | 3 (0.1) |
| Human polyomavirus WU/KI | 0 (0.0) | 0 (0.0) | 8 (5.5) | 13 (0.4) |
| Human bocavirus-1 | 1 (0.7) | 1 (0.1) | 7 (4.8) | 7 (0.2) |
| Adenovirus | 0 (0.0) | 0 (0.0) | 10 (6.9) | 9 (0.3) |
| *Streptococcus pneumoniae* | 72 (49.3) | 268 (15.1) | 101 (69.2) | 556 (17.5) |
| *Moraxella catarrhalis* | 41 (28.1) | 212 (12.0) | 76 (52.1) | 481 (15.1) |
| *Haemophilus influenzae* | 16 (10.9) | 45 (2.5) | 33 (22.6) | 124 (3.9) |
| **Number of children with +ve swabs^a^** | 117 (80.1) |  | 137 (93.8) |  |

^a^Number of ORChID children with at least one positive swab for the virus or potentially pathogenic bacteria of interest.

**E-Table 3: Number and percentages of swabs with rhinovirus detections and episodes with symptoms (N=151 swabs; 71 of 146 children with at least one rhinovirus detection)**

|  | **Detections** | **Episodes^a^** | **Symptoms**  **Number (% of total episodes)** | | | |
| --- | --- | --- | --- | --- | --- | --- |
|  |  |  | **Asymptomatic** | **ARI** | **URI** | **ALRI** |
|  | **Number (%)** | **Number (%)** |  |  |  |  |
| **Rhinovirus** | 151 | 91 | 70 (76.9) | 21 (23.1) | 17 (18.7) | 4 (4.4) |
| **A** | 40 (26.5) | 28 (30.8) | 19 (67.9) | 9 (32.1) | 7 (25.0) | 2 (7.1) |
| **B** | 24 (15.9) | 12 (13.2) | 12 (100.0) | 0 (0.0) | 0 (0.0) | 0 (0.0) |
| **C** | 37 (24.5) | 34 (37.4) | 26 (76.5) | 8 (23.5) | 7 (20.6) | 1 (2.9) |
| **Untyped** | 50 (33.1) | 17 (18.7) | 13 (76.5) | 4 (23.5) | 3 (17.7) | 1 (5.9) |

Abbreviations: ALRI: acute lower respiratory infection; ARI: acute respiratory infection; URI: upper respiratory infection.

^a^A new rhinovirus episode was defined as detecting either a rhinovirus genotype for the first time or, if the same rhinovirus genotype had been detected previously, at least 30-days after the last positive swab for that genotype. Untyped detection was renamed if it was sandwiched by the same type within 30-days. A rhinovirus episode was classified as symptomatic if symptoms were first detected in the 7-days prior to the first virus detection for that episode, or if symptoms were present in the 7-days after the first virus detection.

**E-Table 4: Association between rhinovirus and potentially pathogenic bacterial detections in the first 3-months of life using simple logistic regression (N=1744 swabs)**

|  | **No PPB detected** | **PPB detected** | **OR** | **95%CI** |
| --- | --- | --- | --- | --- |
|  | **N (%)** | **N (%)** |  |  |
|  | *Streptococcus pneumoniae* | |  |  |
| No rhinovirus detections | 1375 (92.7) | 221 (84.7) | Ref |  |
| Rhinovirus detections | 108 (7.3) | 40 (15.3) | **2.30** | **1.56-3.40** |
|  | *Moraxella catarrhalis* | |  |  |
| No rhinovirus detections | 1425 (92.8) | 171 (81.8) | Ref |  |
| Rhinovirus detections | 110 (7.2) | 38 (18.2) | **3.88** | **1.93-4.30** |
|  | *Haemophilus influenzae* | |  |  |
| No rhinovirus detections | 1565 (92.1) | 31 (70.5) | Ref |  |
| Rhinovirus detections | 135 (7.9) | 13 (29.6) | **4.86** | **2.48-9.51** |

Abbreviations: CI: confidence interval; OR: odds ratio; PPB: potentially pathogenic bacteria.

**E-Table 5: Rhinovirus and potentially pathogenic bacterial detections in the first 3-months of life and risk of wheezing (ever) in the first 2-years of life (N=146).**

| Pathogens | No wheeze (ever)  N=98 | Wheeze (ever)  N=48 | Single pathogen Model  N=146 | Single Pathogen Adjusted Model^c^  N=144 |
| --- | --- | --- | --- | --- |
|  | **Mean (SD)**^a^ | **Mean (SD)**^a^ | **IRR (95%CI)** | **IRR (95%CI)** |
| Rhinovirus | 0.82 (1.45) | 1.48 (1.94) | 1.16 (1.00-1.35) | 1.15 (0.96-1.37) |
| *S. pneumoniae* | 1.64 (2.64) | 2.02 (2.99) | 1.04 (0.94-1.15) | 1.04 (0.93-1.16) |
| *M. catarrhalis* | 1.35 (3.04) | 1.56 (3.13) | 1.02 (0.94-1.12) | 1.02 (0.92-1.14) |
| *H. influenzae* | 0.27 (0.99) | 0.35 (1.23) | 1.08 (0.85-1.36) | 1.08 (0.84-1.39) |

Abbreviations: CI: confidence interval; IRR: incident rate ratio; SD: standard deviation.

^a^Mean number of weeks a pathogen was detected in the first 3-months of life. ^b^A single poisson regression analysis with four main effects (rhinovirus, *S. pneumoniae, M. catarrhalis*, and *H. influenzae*). ^c^Adjusted for season of birth, maternal asthma history, gestational age, delivery method, tobacco smoke exposure at birth, exclusive breastfeeding during the first 3-months of life, and older child in the household at birth. Childcare attendance at 3-months was omitted as there were only four children attending any form of childcare.

**E-Table 6: Rhinovirus and potentially pathogenic bacterial detections in the first 4-weeks and 6-months of life, and risk of wheezing (ever) in the first 2-years of life (N=146)****.**

| Pathogens |  | No Wheeze  (ever)  N=98 | Wheeze  (ever)  N=48 | Single pathogen Model  N=146 | Rhinovirus and bacteria Model  N=146^b^ | Single Pathogen Adjusted Model^c^  N= 143 | Full Adjusted Model^c^  N=143 |
| --- | --- | --- | --- | --- | --- | --- | --- |
|  |  | **Mean (SD)** ^a^ | **Mean (SD)** ^a^ | **IRR (95% CI)** | **IR (95% CI)** | **IRR (95% CI)** | **IRR (95% CI)** |
| Pathogens in the first  4-weeks of life | Rhinovirus | 0.15 (0.50) | 0.15 (0.50) | 0.81 (0.52–1.62) | 0.90 (0.50-1.63) | 0.91 (0.50-1.63) | 0.89 (0.49-1.65) |
|  | *S. pneumoniae* | 0.35 (0.72) | 0.35 (0.79) | 1.00 (0.69–1.46) | 1.00 (0.69-1.47) | 0.98 (0.64-1.51) | 0.98 (0.63-1.53) |
|  | *M. catarrhalis* | 0.23 (0.67) | 0.21 (0.65) | 1.02 (0.66–1.59) | 1.04 (0.66-1.65) | 1.03 (0.62-1.70) | 1.06 (0.63–1.79) |
|  | *H. influenzae* | 0.02 (0.14) | 0.02 (0.14) | 0.96 (0.13–6.96) | 0.95 (0.13-6.96) | 1.14 (0.14-9.51) | 1.15 (0.13–9.83) |
| Pathogens in the first  6-months of life | Rhinovirus | 2.55 (2.49) | 4.52 (3.90) | **1.11 (1.03–1.19)** | **1.11 (1.04–1.20)** | **1.12 (1.03-1.23)** | **1.13 (1.04–1.24)** |
|  | *S. pneumoniae* | 3.79 (5.03) | 5.02 (5.64) | 1.03 (0.98–1.08) | 1.02 (0.96–1.08) | 1.02 (0.96-1.08) | 1.02 (0.95–1.08) |
|  | *M. catarrhalis* | 3.35 (5.61) | 3.73 (5.26) | 1.01 (0.96–1.06) | 0.99 (0.94–1.05) | 1.01 (0.95-1.07) | 1.02 (0.96–1.08) |
|  | *H. influenzae* | 0.97 (2.29) | 0.92 (2.47) | 1.00 (0.89–1.13) | 0.94 (0.83–1.07) | 0.98 (0.87-1.11) | 0.94 (0.83–1.08) |

Abbreviations: CI: confidence interval; IRR: Incident rate ratio; SD: standard deviation.

^a^Mean number of weeks a pathogen was detected during the time-period of interest. ^b^A single poisson regression analysis with four main effects (rhinovirus, *S. pneumoniae, M. catarrhalis*, and *H. influenzae*). ^c^Adjusted for season of birth, maternal asthma history, gestational age, delivery method, tobacco smoke exposure at birth, exclusive breastfeeding during the first 3-months of life, and older child in the household at birth. Childcare attendance at 3-months was omitted as there were only four children attending any form of childcare. Childcare attendance at
6-months was added to the 6-months model.

**E-Table 7: Rhinovirus species and potentially pathogenic bacterial detections in the first 3-months of life and risk of wheeze (ever) in the first 2-years of life (N=146).**

| Pathogens |  | No Wheeze (ever)  N=98 | Wheeze (ever)  N=48 | Single pathogen  Model  N=146 | Rhinovirus and Bacteria Model ^b^  N=146 | Single Pathogen Adjusted Model^c^  N=144 | Full Adjusted Model^c^  N=144 |
| --- | --- | --- | --- | --- | --- | --- | --- |
|  |  | **Mean (SD)**^a^ | **Mean (SD)**^a^ | **IRR (95% CI)** | **IRR (95% CI)** | **IRR (95% CI)** | **IRR (95% CI)** |
| Rhinovirus A | Rhinovirus-A | 0.34 (0.94) | 0.39 (0.94) | 1.02 (0.77–1.34) | 0.98 (0.72–1.33) | 0.90 (0.62-1.32) | 0.88 (0.59–1.31) |
|  | *S. pneumoniae* | 1.64 (2.64) | 2.02 (2.99) | 1.04 (0.94–1.15) | 1.03 (0.92–1.16) | 1.04 (0.93-1.16) | 1.03 (0.91–1.17) |
|  | *M. catarrhalis* | 1.35 (3.03) | 1.56 (3.13) | 1.02 (0.94–1.12) | 1.01 (0.92–1.12) | 1.02 (0.92-1.14) | 1.02 (0.91–1.14) |
|  | *H. influenzae* | 0.27 (0.99) | 0.35 (1.23) | 1.08 (0.85–1.36) | 1.03 (0.78–1.35) | 1.08 (0.84-1.39) | 1.03 (0.77–1.39) |
| Rhinovirus B | Rhinovirus-B | 0.13 (0.79) | 0.31 (1.24) | 1.14 (0.91–1.43) | 1.13 (0.91–1.43) | 1.09 (0.86-1.40) | 1.10 (0.86–1.41) |
|  | *S. pneumoniae* | 1.64 (2.64) | 2.02 (2.98) | 1.04 (0.94–1.15) | 1.02 (0.92–1.15) | 1.04 (0.93-1.16) | 1.02 (0.90–1.16) |
|  | *M. catarrhalis* | 1.35 (3.03) | 1.56 (3.13) | 1.02 (0.94–1.12) | 1.01 (0.92–1.11) | 1.02 (0.92-1.14) | 1.02 (0.91–1.14) |
|  | *H. influenzae* | 0.27 (0.99) | 0.35 (1.23) | 1.08 (0.85–1.36) | 1.03 (0.78–1.36) | 1.08 (0.84-1.39) | 1.04 (0.78–1.40) |
| Rhinovirus C | Rhinovirus-C | 0.23 (0.61) | 0.63 (1.47) | 1.22 (0.99–1.48) | 1.21 (0.98–1.49) | 1.19 (0.97-1.49) | 1.19 (0.96–1.48) |
|  | *S. pneumoniae* | 1.64 (2.64) | 2.02 (2.98) | 1.04 (0.94–1.15) | 1.03 (0.92–1.16) | 1.03 (0.93-1.16) | 1.03 (0.91–1.17) |
|  | *M. catarrhalis* | 1.35 (3.03) | 1.56 (3.13) | 1.02 (0.94–1.12) | 0.99 (0.90–1.10) | 1.02 (0.92-1.14) | 1.00 (0.90–1.13) |
|  | *H. influenzae* | 0.27 (0.99) | 0.35 (1.23) | 1.08 (0.85–1.36) | 1.00 (0.76–1.33) | 1.08 (0.84-1.39) | 1.01 (0.76–1.37) |

Abbreviations: CI: confidence interval; IRR: incident rate ratio; SD: standard deviation.

^a^Mean number of weeks a pathogen was detected in the first 3-months of life. ^b^A single poisson regression analysis with four main effects (rhinovirus, *S. pneumoniae, M. catarrhalis*, and *H. influenzae*). ^c^Adjusted for season of birth, maternal asthma history, gestational age, delivery method, tobacco smoke exposure at birth, exclusive breastfeeding during the first 3-months of life, and older child in the household at birth. Childcare attendance at 3-months was omitted as there were only four children attending any form of childcare.

**E-Table 8: Rhinovirus and potentially pathogenic bacterial detections in the first 3-months of life and asthma (ever) at ages 5–7-years (N=84).**

| Pathogen | No asthma (ever)  N=55 | Asthma (ever)  N=29 | Single pathogen Model  N=84 | Single Pathogen Adjusted Model^c^  N=84 |
| --- | --- | --- | --- | --- |
|  | **Mean (SD)**^a^ | **Mean (SD)**^a^ | **OR (95%CI)** | **OR (95%CI)** |
| Rhinovirus | 0.98 (1.58) | 1.07 (1.46) | 1.04 (0.78–1.39) | 1.12 (0.77-1.63) |
| *S. pneumoniae* | 1.65 (2.57) | 1.21 (2.13) | 0.92 (0.75–1.13) | 0.90 (0.71-1.15) |
| *M. catarrhalis* | 1.44 (2.98) | 1.66 (3.65) | 1.02 (0.89–1.17) | 1.12 (0.91-1.36) |
| *H. influenzae* | 0.07 (0.33) | 0.45 (1.43) | 1.74 (0.81–3.76) | 1.74 (0.79-3.83) |

Abbreviations: CI: confidence interval; OR: odds ratio; SD: standard deviation.

^a^Mean number of weeks a pathogen was detected in the first 3-months of life. ^b^A single logistic regression analysis with four main effects (rhinovirus, *S. pneumoniae, M. catarrhalis*, and *H. influenzae*). ^c^Adjusted for season of birth, parental asthma history, gestational age, delivery method, tobacco smoke exposure at birth, exclusive breastfeeding during the first 3-months of life, and older child in the household at birth. Childcare attendance at 3-months was omitted as there were only four children attending any form of childcare.

**E-Table 9: Rhinovirus and potentially pathogenic bacterial detections in the first 4-weeks and 6-months of life and the risk of asthma (ever) at ages 5–7-years (N=84)****.**

| Pathogens |  | No Asthma  (ever)  N=55 | Asthma  (ever)  N=29 | Single pathogen Model  N=84 | Rhinovirus and Bacteria Model^b^  N=84 | Single Pathogen Adjusted Model^c^  N=84 | Full Adjusted Model^c^  N=84 |
| --- | --- | --- | --- | --- | --- | --- | --- |
|  |  | **Mean (SD)** ^a^ | **Mean (SD)** ^a^ | **OR (95%CI)** | **OR (95%CI)** | **OR (95%CI)** | **OR (95%CI)** |
| Pathogens  in the first  4-weeks of life | Rhinovirus | 0.11 (0.46) | 0.17 (0.47) | 1.33 (0.52–3.43) | 1.45 (0.53–3.97) | 1.12 (0.77-1.63) | 1.49 (0.46–4.80) |
|  | *S. pneumoniae* | 0.36 (0.80) | 0.31 (0.66) | 0.91 (0.49–1.68) | 0.84 (0.43–1.65) | 0.90 (0.71-1.15) | 0.67 (0.29–1.52) |
|  | *M. catarrhalis* | 0.24 (0.72) | 0.24 (0.58) | 1.01 (0.52–1.98) | 1.00 (0.50–2.00) | 1.11 (0.91-1.36) | 1.50 (0.56–4.02) |
|  | *H. influenzae* | 0.02 (0.13) | 0 (0.0) | N.A | N.A | N.A | N.A |
| Pathogens in the first  6-months of life | Rhinovirus | 3.33 (3.56) | 3.17 (3.05) | 0.99 (0.86–1.13) | 0.96 (0.83–1.12) | 1.12 (0.77-1.63) | 1.00 (0.82–1.22) |
|  | *S. pneumoniae* | 4.04 (4.77) | 3.93 (5.56) | 1.00 (0.91–1.09) | 0.97 (0.87–1.08) | 1.01 (0.91-1.13) | 0.96 (0.85–1.09) |
|  | *M. catarrhalis* | 3.45 (5.37) | 3.34 (5.65) | 1.00 (0.92–1.08) | 0.98 (0.90–1.08) | 1.05 (0.93-1.18) | 1.02 (0.89–1.16) |
|  | *H. influenzae* | 0.67 (1.67) | 1.45 (3.65) | 1.12 (0.94–1.34) | 1.18 (0.96–1.45) | 1.25 (0.99-1.58) | 1.27 (0.98–1.65) |

Abbreviations: CI: confidence interval; NA: Not applicable; OR: odds ratio; SD: standard deviation.

^a^Mean number of weeks a pathogen was detected during the time-period of interest. ^b^A single logistic regression analysis with four main effects (rhinovirus, *S. pneumoniae, M. catarrhalis*, and *H. influenzae*). ^c^Adjusted for season of birth, parental asthma history, gestational age, delivery method, tobacco smoke exposure at birth, and older child in the household at birth. Childcare attendance at 3-months was omitted as there were only four children attending any form of childcare. Childcare attendance at 6-months was added to the 6-months model.

**E-Table 10: Rhinovirus species and potentially pathogenic bacterial detections in the first 3-months of life and the risk of asthma (ever) at ages 5–7-years (N=84).**

| Pathogens |  | No Asthma (ever)  N=55 | Asthma  (ever)  N=29 | Single pathogen Model  N=84 | Rhinovirus and bacteria Model ^b^  N=84 | Single Pathogen Adjusted Model^c^  N=84 | Full Adjusted Model^c^  N=84 |
| --- | --- | --- | --- | --- | --- | --- | --- |
|  |  | **Mean (SD)**^a^ | **Mean (SD)**^a^ | **OR (95%CI)** | **OR (95%CI)** | **OR (95%CI)** | **OR (95%CI)** |
| Rhinovirus A | Rhinovirus-A | 0.38 (0.97) | 0.45 (0.95) | 1.07 (0.68–1.70) | 1.04 (0.62–1.74) | 1.35 (0.74-2.47) | 1.38 (0.72–2.64) |
|  | *S. pneumoniae* | 1.65 (2.57) | 1.21 (2.13) | 0.92 (0.75–1.13) | 0.77 (0.58–1.04) | 0.90 (0.71-1.15) | 0.71 (0.48–1.04) |
|  | *M. catarrhalis* | 1.44 (2.98) | 1.66 (3.65) | 1.02 (0.89–1.17) | 0.99 (0.84–1.16) | 1.11 (0.91-1.36) | 1.09 (0.86–1.38) |
|  | *H. influenzae* | 0.07 (0.33) | 0.45 (1.43) | 1.75 (0.81–3.76) | **2.39 (1.00–5.78)** | 1.74 (0.79-3.83) | 2.27 (0.87–5.97) |
| Rhinovirus B | Rhinovirus-B | 0.16 (0.86) | 0.28 (1.16) | 1.12 (0.72–1.75) | 1.08 (0.69–1.70) | 1.25 (0.75-2.07) | 1.16 (0.69–1.93) |
|  | *S. pneumoniae* | 1.65 (2.57) | 1.21 (2.13) | 0.92 (0.75–1.13) | 0.78 (0.58–1.05) | 0.93 (0.72-1.19) | 0.74 (0.51–1.06) |
|  | *M. catarrhalis* | 1.44 (2.98) | 1.66 (3.65) | 1.02 (0.89–1.17) | 0.99 (0.85–1.17) | 0.97 (0.75-1.27) | 1.10 (0.87–1.41) |
|  | *H. influenzae* | 0.07 (0.33) | 0.45 (1.43) | 1.74 (0.81–3.76) | **2.38 (1.00–5.72)** | 1.80 (0.80-4.04) | 2.22 (0.88–5.61) |
| Rhinovirus C | Rhinovirus-C | 0.38 (1.13) | 0.14 (0.35) | 0.66 (0.30–1.45) | 0.59 (0.23–1.53) | 0.66 (0.31-1.42) | 0.56 (0.21–1.48) |
|  | *S. pneumoniae* | 1.65 (2.57) | 1.21 (2.13) | 0.92 (0.75–1.13) | 0.77 (0.57–1.04) | 0.90 (0.71-1.15) | 0.72 (0.50–1.04) |
|  | *M. catarrhalis* | 1.44 (2.97) | 1.66 (3.65) | 1.02 (0.89–1.17) | 0.99 (0.85–1.18) | 1.11 (0.91-1.36) | 1.10 (0.86–1.40) |
|  | *H. influenzae* | 0.07 (0.33) | 0.45 (1.43) | 1.74 (0.81–3.76) | **2.46 (1.02–5.92)** | 1.74 (0.80-3.83) | 2.38 (0.94–5.99) |

Abbreviations: CI: confidence interval; OR: odds ratio; SD: standard deviation.

^a^Mean number of weeks a pathogen was detected in the first 3-months of life. ^b^A single logistic regression analysis with four main effects (rhinovirus, *S. pneumoniae, M. catarrhalis*, and *H. influenzae*). ^c^Adjusted for season of birth, parental asthma history, gestational age, delivery method, tobacco smoke exposure at birth, and older child in the household at birth. Childcare attendance at 3-months was omitted as there were only four children attending any form of childcare.

**E-Image 1**: **Directed acyclic graph for impact of rhinovirus and potentially pathogenic bacteria in the first 3-months of life on wheeze in the first 2-years of life.**


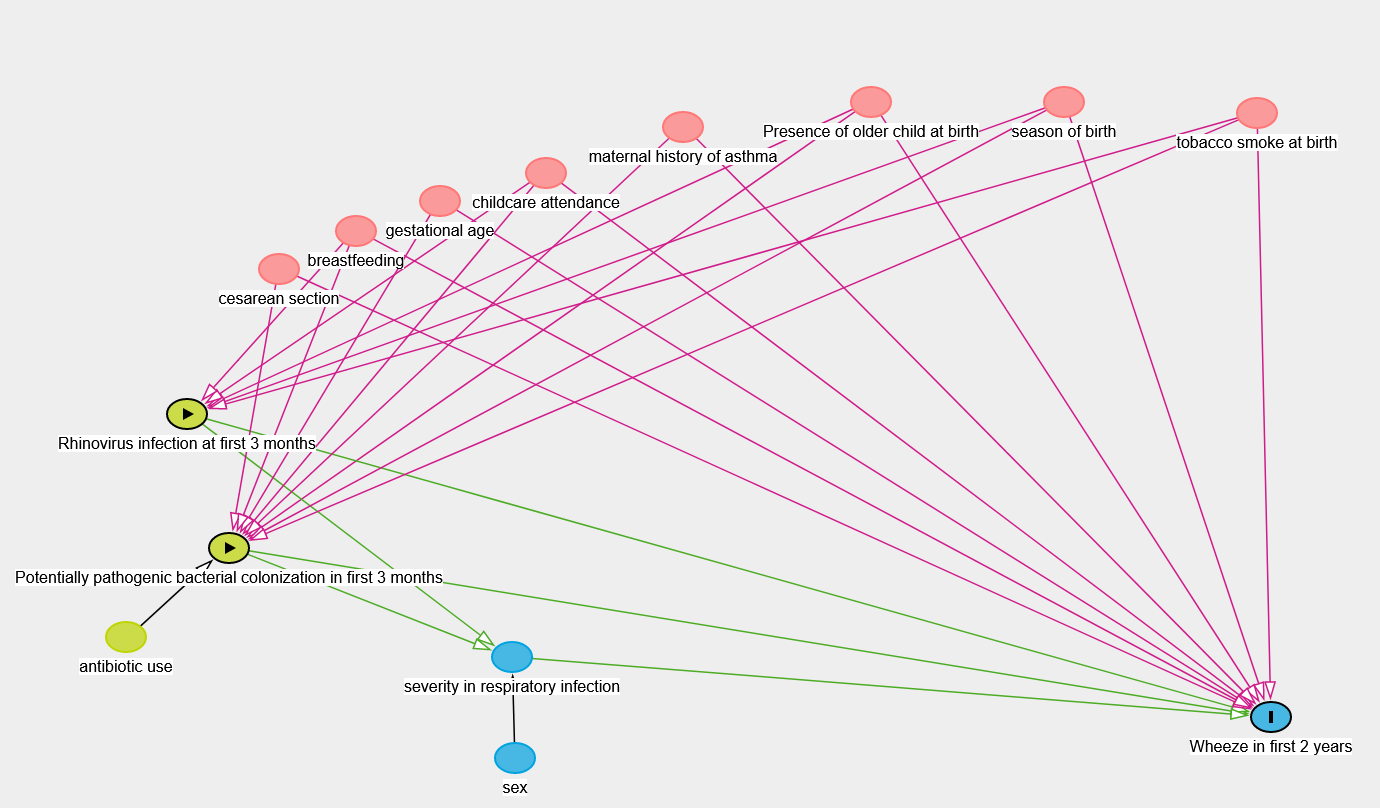


| **Legend** |
| --- |
| 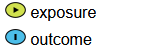  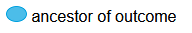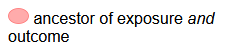 |

**E-Image 2**: **Directed acyclic graph for impact of rhinovirus and potentially pathogenic bacteria in the first 3-months of life on asthma at ages 5–7-years.**


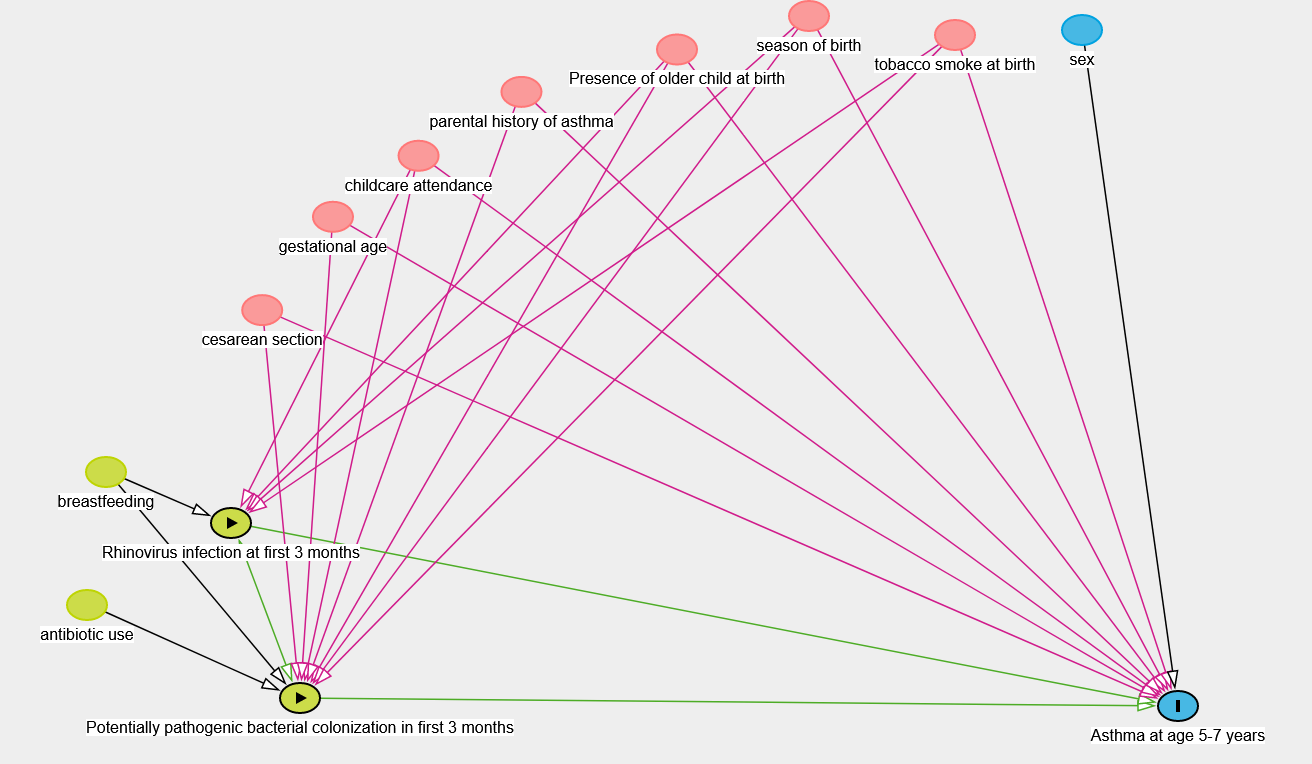


| **Legend** |
| --- |
| 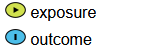  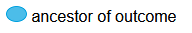  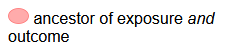 |

**E-Image 3: Proportion of swab and/or diary returns for 146 participants in the ORChID study in the first 6-months of life.**


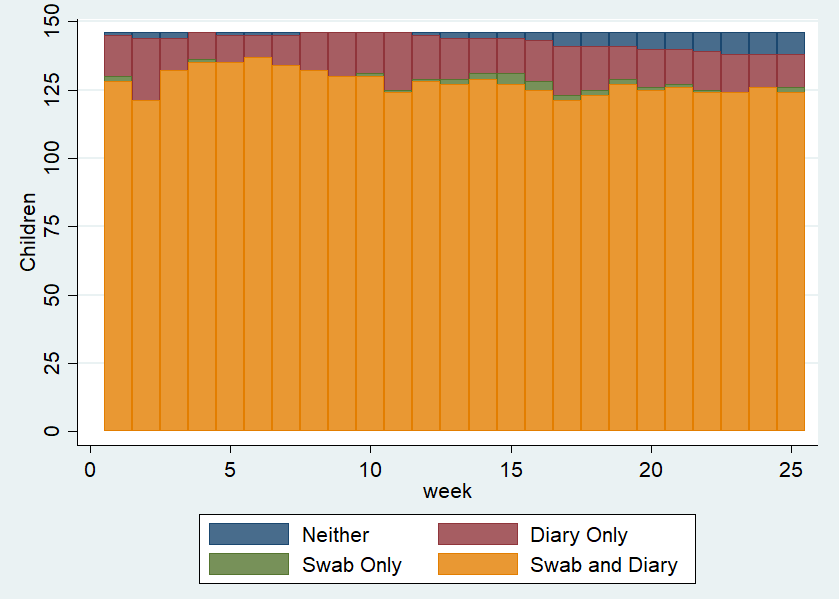


**E-Image 4: Number of detections of rhinovirus and** **potentially pathogenic bacteria for 146 participants in the ORChID study in the first 6-months of life.**

Rhinovirus, *S. pneumoniae, M. catarrhalis*, and *H. influenzae* were detected at least once in 71 (48.6%), 72 (49.3%), 41 (28.1%), and 16 (10.9%) cohort children, respectively during the same period.

**E-Image 5:** **Weeks of rhinovirus and potentially pathogenic bacterial detections in the first 3-months of life and probability of wheeze (ever) in the first 2-years of life (N=146)**


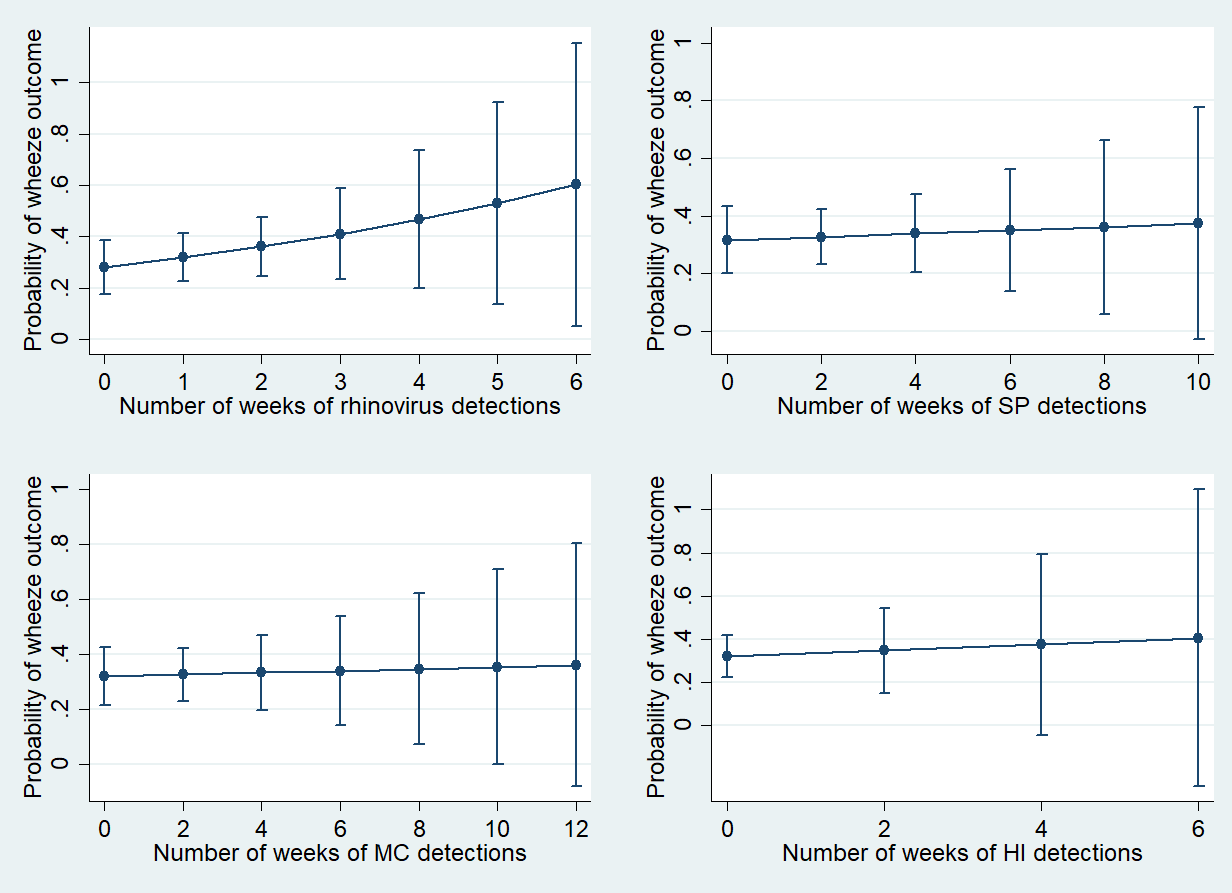


Abbreviations: HI: *Haemophilus influenzae*; MC: *Moraxella catarrhalis*; SP: *Streptococcus pneumoniae*

The probability of an outcome at the nominated week was derived from the marginal effects, which is a way of presenting results as differences in probabilities for the nominated week. Adjusted for season of birth, maternal asthma history, gestational age, delivery method, tobacco smoke exposure at birth, exclusive breastfeeding during the first 3-months of life, and older child in the household at birth. Childcare attendance at 3-months was omitted as there were only four children attending any form of childcare.

**E-Image 6: Weeks of rhinovirus and potentially pathogenic bacterial detections in the first 3-months of life and probability of asthma (ever) at ages 5–7-years (N=84)**


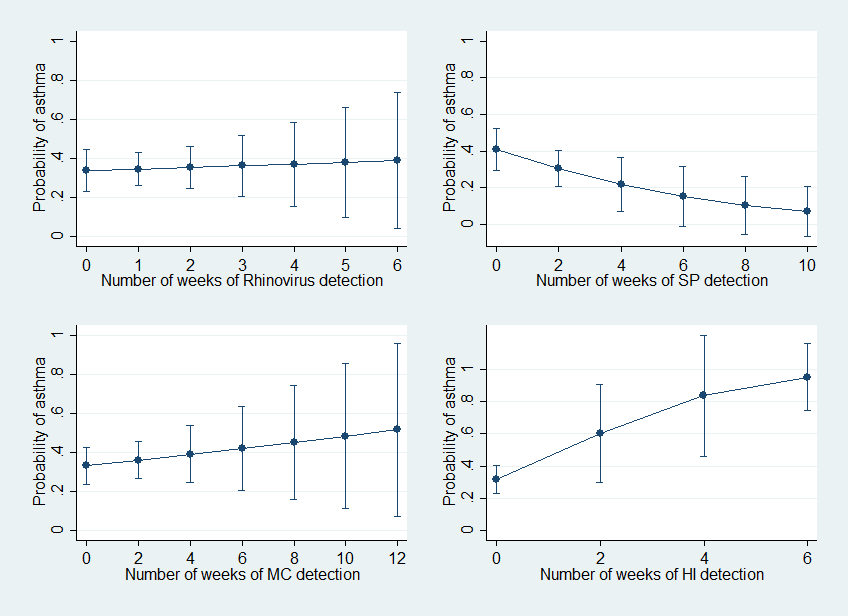


Abbreviations: CI: confidence interval; HI: *Haemophilus influenzae*; MC: *Moraxella catarrhalis*; SP: *Streptococcus pneumoniae*

The probability of an outcome at the nominated week was derived from the marginal effects, which is a way of presenting results as differences in probabilities for the nominated week. Adjusted for season of birth, parental asthma history, gestational age, delivery method, tobacco smoke exposure at birth, exclusive breastfeeding during the first 3-months of life, and older child in the household at birth. Childcare attendance at 3-months was omitted as there were only four children attending any form of childcare.
